# Phased rehabilitation nursing improves hip function, reduces complications, and enhances quality of life following total hip arthroplasty: A prospective quasi-experimental cohort study

**DOI:** 10.1097/MD.0000000000049093

**Published:** 2026-07-10

**Authors:** Xin Wang, Dan Wang

**Affiliations:** a Department of Orthopedics, Ankang People’s Hospital, Ankang, Shaanxi, China; b Department of Surgical Anesthesia, Ankang People’s Hospital, Ankang, Shaanxi, China.

**Keywords:** exercise compliance, hip function, pain self-care ability, phased rehabilitation nursing, quasi-experimental study, total hip arthroplasty

## Abstract

Total hip arthroplasty (THA) is the definitive treatment for advanced hip disease; however, postoperative rehabilitation remains challenging, particularly in elderly patients with multiple comorbidities. This study evaluated the effectiveness of phased rehabilitation (PR) nursing versus routine care on recovery outcomes following THA. This prospective quasi-experimental cohort study included 100 patients who underwent primary unilateral THA at Ankang People’s Hospital from January 2023 to December 2024 and were assigned to either a study group (n = 50) receiving a 3-phase rehabilitation nursing protocol or a control group (n = 50) receiving routine postoperative care. Group allocation was determined by ward assignment and nursing team availability rather than formal randomization. The phased protocol included structured progression from early muscle stimulation and mobilization (phase 1) to functional transition training (phase 2) and advanced mobility training (phase 3). Primary outcomes were pain intensity (Visual Analog Scale) and hip function (Harris Hip Score). Secondary outcomes included quality of life (Short-Form 36), complications, and patient satisfaction, assessed at 3 and 6 months postoperatively. Repeated-measures analysis of variance was employed to evaluate longitudinal changes, supplemented by pairwise comparisons at each time point. At 6 months, the PR group demonstrated significantly lower pain scores (Visual Analog Scale) and superior hip function (Harris Hip Score) compared with the control group (*P* < .05). Repeated-measures analysis of variance confirmed significant time-by-group interaction effects for primary outcomes (all *P* < .05). The intervention group also had a significantly lower overall complication rate (2.0% vs 16.0%, *P* = .01), although the small absolute number of events warrants cautious interpretation. Furthermore, patients who received PR reported significantly better outcomes across all 8 domains of the Short-Form 36 quality of life survey, greater functional independence (Barthel Index), higher self-care capacity (Exercise of Self-care Agency Scale), better exercise compliance (96% vs 80%), and higher patient satisfaction (94% vs 78%; all *P* < .05). PR nursing significantly enhances recovery after THA through systematic, progressive interventions tailored to recovery stages, thereby reducing complications and improving functional outcomes, quality of life, and patient satisfaction. These findings, derived from a single-center quasi-experimental design, require confirmation through multicenter randomized controlled trials.

## 1. Introduction

Hip diseases, including fractures, arthritis, avascular necrosis, congenital dysplasia, and rheumatoid conditions, are increasingly prevalent due to an aging population, lifestyle factors, trauma, alcohol use, corticosteroid therapy, obesity, and metabolic changes.^[1]^ Total hip arthroplasty (THA) remains the definitive treatment for advanced hip disease causing pain, stiffness, and functional impairment.^[2]^ This surgical procedure, which involves replacing damaged hip joints with prosthetic components, has evolved over a century of refinement.^[3]^ Continuous improvements in surgical techniques and implant materials have expanded the indications and increased the number of THA procedures performed annually.^[4]^

The increasing number of THA surgeries has increased the demand for effective postoperative (PO) rehabilitation.^[5]^ However, rehabilitation after THA faces significant challenges. Many patients are elderly with multiple comorbidities, limited baseline physical function, and reduced exercise capacity.^[6,7]^ PO pain and limb length discrepancy can further complicate recovery, making targeted rehabilitation essential.^[8]^

Evidence demonstrates that early functional exercise after THA reduces complications and improves quality of life.^[9]^ However, hip function recovery requires sustained, progressive rehabilitation over extended periods to achieve optimal outcomes.^[10]^ Phased rehabilitation (PR) nursing represents a patient-centered approach that tailors interventions to specific recovery stages based on individual patient needs and capabilities.^[11]^ This model has shown promise in accelerating recovery and improving outcomes after percutaneous coronary intervention.^[12,13]^ However, its effectiveness in THA rehabilitation remains unexplored.

Therefore, this study aimed to evaluate the effects of PR nursing on hip function recovery and quality of life in patients undergoing THA.

## 2. Methods

### 2.1. Study design and setting

This prospective quasi-experimental cohort study was conducted at the Department of Orthopedics, Ankang People’s Hospital, from January 2023 to December 2024. The study aimed to evaluate the comparative effectiveness of PR nursing versus routine nursing care in patients undergoing THA. The quasi-experimental design was selected to evaluate the PR intervention under real-world clinical conditions, with group allocation determined by ward assignment and the availability of nursing teams trained in the PR protocol at the time of each patient’s admission. This pragmatic allocation approach reflected routine clinical operations, wherein patients were admitted to available wards based on bed availability rather than clinical characteristics, and wards staffed by trained rehabilitation nurses constituted the study group, while the remaining wards served as the control group.

This study was approved by the Ankang People’s Hospital Medical Ethics Committee. All procedures adhered to the principles of the Declaration of Helsinki for medical research involving human subjects. Written informed consent was obtained from all participants or their legally authorized representatives prior to enrollment. Participants retained the right to withdraw from the study at any time without affecting standard clinical care.

### 2.2. Participants

#### 2.2.1. Sample size and recruitment

We recruited 100 consecutive patients who underwent THA at our hospital during the study period. Patients were approached for participation during their preoperative consultation, and written informed consent was obtained prior to surgery. Participants were assigned to either the control group or the study group based on ward assignment at the time of admission, which was governed by bed availability and nursing team allocation rather than patient clinical characteristics. Fifty patients were allocated to each group. Baseline demographic and clinical characteristics were compared between groups to assess comparability (Table 1), and no statistically significant differences were identified, supporting the adequacy of this allocation approach for the purposes of the present study.

**Table 1:** Baseline demographic and clinical characteristics of study participants.

Characteristics	Control group (n = 50)	Study group (n = 50)	Statistical value	*P*-value
Sex, n (%)			χ^2^ = 0.04	.84
Male	28 (56.0)	27 (54.0)		
Female	22 (44.0)	23 (46.0)		
Age, yr			*t* = 0.11	.90
Mean ± SD	69.61 ± 3.32	69.53 ± 3.45		
Primary diagnosis, n (%)			χ^2^ = 0.05	.97
Femoral neck fracture	25 (50.0)	26 (52.0)		
Avascular necrosis of femoral head	15 (30.0)	14 (28.0)		
Hip osteoarthritis	10 (20.0)	10 (20.0)		

Data are presented as n (%) for categorical variables and mean ± standard deviation for continuous variables. *P*-values derived from independent samples *t* tests for continuous variables and chi-square tests for categorical variables.

SD = standard deviation.

### 2.3. Inclusion and exclusion criteria

Participants were eligible for inclusion if they met the following criteria:

1. Confirmed diagnosis of hip pathology requiring THA through imaging examination and clinical assessment.
2. Scheduled for primary unilateral THA.
3. Age ≥ 18 years.

Patients were excluded if they presented with the following:

1. Concurrent malignant neoplasm.
2. Previous orthopedic surgery on the affected hip.
3. Severe mental illness or cognitive impairment that would preclude participation in rehabilitation protocols.

Additional exclusion criteria included bilateral hip disease requiring simultaneous surgery, revision arthroplasty, and inability to provide informed consent.

### 2.4. Interventions

#### 2.4.1. Control group: routine nursing care

The control group received standard PO nursing care following established hospital protocols. This comprehensive care included medication administration according to physician orders, with regular monitoring of the operative limb for changes in skin temperature, color, and circulation. The patients received standard positioning care with scheduled repositioning assistance to prevent pressure complications. Oral hygiene and skin care were performed twice daily, while surgical site management involved regular dressing changes with strict incision protection protocols to prevent wound trauma. Pain management followed standard institutional protocols using multimodal analgesia. Early mobilization was encouraged based on physician assessment, with activity levels tailored to individual patient tolerance.

#### 2.4.2. Study group: PR nursing protocol

The intervention group received a structured PR nursing protocol developed through interdisciplinary collaboration between the nursing, orthopedic surgery, and rehabilitation medicine departments. The protocol was standardized through written clinical guidelines, and all participating nursing staff completed a structured 16-hour training program prior to study initiation. This training program encompassed theoretical instruction on PO rehabilitation principles, hands-on technique workshops supervised by rehabilitation medicine specialists, and competency assessments that all nurses were required to pass before independently delivering the intervention. Protocol fidelity was monitored through weekly supervisory audits of nursing documentation, periodic direct observations of rehabilitation sessions by the research team, and standardized treatment logs completed after each session. Any protocol deviations identified were addressed during weekly interdisciplinary team meetings.

Phase 1 (PO days 1–4): Upon return to the ward, patients received targeted muscle stimulation therapy involving manual systematic compression of the bilateral lower extremities by trained nursing staff, progressing from distal-to-proximal segments, including the gastrocnemius, quadriceps femoris, and biceps femoris muscles. Each compression session was standardized at 15 minutes in duration, performed thrice daily, with moderate pressure (sufficient to produce visible tissue blanching without patient discomfort) applied in a systematic distal-to-proximal sequence. Following anesthesia recovery, patients were positioned laterally with appropriate limb support and initiated ankle flexion-extension exercises for 10 minutes, performed 2 to 3 times daily. Progressive foot flexion-extension training commenced within 72 hours postoperatively, maintaining hip motion within a 30° angle. On PO day 4, mechanical knee joint exercise equipment was introduced with gradual range-of-motion progression from 0° to 30°, increasing by 10° increments during 10-minute sessions performed 2 to 3 times daily.

Phase 2 (PO days 5–7): This phase emphasized functional transition training, incorporating limb extension exercises and progressive position changes. Hip abduction exercises were performed at graduated angles (5°, 10°, 15°, and 20°) with 10-second hold times during 10 to 15-minute sessions. Position transition training progressed from semi-recumbent to sitting positions with careful monitoring for orthostatic symptoms. Upon successful tolerance, patients initiated standing and gait training using walker assistance for 10-minute sessions 3 times daily. Therapeutic heat application using Teding Diancibo Pu (special electromagnetic spectrum) lamp therapy was introduced on PO day 5 and administered for 30 minutes 3 times daily to promote local circulation and tissue healing.

Phase 3 (beginning PO week 2): Advanced mobility training commenced with walker-assisted ambulation, progressing to bilateral crutch use. Patients practiced coordinated movement patterns with appropriate weight distribution and gait mechanics. Training progression included flat surface ambulation advancing to stair navigation, with specific techniques for ascending (healthy limb leading) and descending (affected limb leading) stairs. Sessions lasted 15 to 20 minutes and were performed 3 times daily under supervision.

### 2.5. Complementary interventions

Nutritional guidance was individualized based on the PO recovery stage. During weeks 1 to 2, emphasis was placed on easily digestible foods with properties that promote circulation and healing. Weeks 3 to 4 focused on calcium-rich foods and adequate protein intake to support bone healing and muscle recovery.

Discharge planning included comprehensive patient education regarding activity restrictions, including maintaining hip flexion below 90°, avoiding crossed-leg positions, and protecting against excessive joint stress for the initial 3 months. Written discharge instructions were provided in patient-friendly language with visual aids demonstrating safe positioning and movement techniques. Post-discharge follow-up was structured with clinic visits every 1 to 2 weeks for the first 3 months, followed by monthly visits through 6 months. Telephone or video follow-up occurred biweekly to monitor adherence and address patient concerns.

### 2.6. Outcome measures

#### 2.6.1. Primary outcomes

Pain intensity was assessed using the Visual Analog Scale (VAS),^[14]^ a validated 10-cm horizontal line where patients marked their current pain level from 0 (no pain) to 10 (worst imaginable pain). The VAS demonstrates excellent test-retest reliability (intraclass correlation coefficient > 0.90) and construct validity in acute pain assessment. Hip function recovery was evaluated using the Harris Hip Score (HHS),^[15]^ a comprehensive assessment tool encompassing pain, deformity, range of motion, and functional capacity domains, with higher scores indicating superior hip function.

#### 2.6.2. Secondary outcomes

Quality of life was assessed using the Short-Form 36 (SF-36) questionnaire,^[16]^ which evaluates 8 health domains, including physical function, bodily pain, role limitations, emotional well-being, social functioning, energy/fatigue, emotional health, and general health perceptions, with scores ranging from 0 to 100. Activities of daily living were measured using the Barthel Index,^[17]^ a 10-item scale assessing independence in self-care and mobility, with a maximum score of 100 points. Self-care capacity was evaluated using the Exercise of Self-care Agency Scale (ESCA),^[18]^ which examines health knowledge, self-concept, self-responsibility, and self-care skills across a 100-point scale.

Complication surveillance included systematic monitoring for joint stiffness (defined as a range of motion less than the functional requirements), deep venous thrombosis (confirmed by Doppler ultrasound when clinically suspected), and pressure injuries (graded according to the National Pressure Injury Advisory Panel guidelines).

Exercise compliance was assessed through a combination of patient self-report exercise diaries and therapist documentation reviewed at each follow-up visit, using a structured questionnaire adapted from established rehabilitation adherence frameworks in the orthopedic literature. Adherence was categorized as complete (strict protocol adherence), partial (occasional protocol deviations with fewer than 2 missed sessions per week), or noncompliant (frequent protocol omissions with 2 or more missed sessions per week). Patient satisfaction with nursing care was evaluated using a validated 100-point institutional questionnaire that had undergone internal consistency testing (Cronbach α > 0.85), covering multiple domains, including pain management, patient education, emotional support, and care coordination. Scores were categorized as very satisfied (≥90 points), satisfied (80–89 points), or dissatisfied (<80 points).

### 2.7. Data collection procedures

Baseline assessments were conducted within 24 hours preoperatively by trained research nurses blinded to group allocation. Follow-up assessments occurred at 3 and 6 months postoperatively during scheduled clinic visits. Data collection forms were standardized, and inter-rater reliability was established through training sessions, achieving agreement coefficients >0.85.

### 2.8. Statistical analysis

Data analysis was performed using Statistical Package for the Social Sciences version 24.0 (IBM Corporation). Continuous variables are expressed as the mean ± standard deviation. For the primary analysis of longitudinal outcomes, repeated-measures analysis of variance (RM-ANOVA) was employed to evaluate time (baseline, 3 months, and 6 months), group (study vs control), and time-by-group interaction effects. The Greenhouse-Geisser correction was applied when the assumption of sphericity was violated, as assessed by the Mauchly test of sphericity. Post hoc pairwise comparisons were conducted using independent samples *t* tests for between-group comparisons and paired *t* tests for within-group changes at each time point. Categorical variables are presented as frequencies and percentages, with group comparisons conducted using the chi-square test or Fisher exact test when expected cell counts were <5. Normality of distribution was verified using the Shapiro–Wilk test. Appropriate nonparametric tests were employed for non-normally distributed data. Statistical significance was defined as *P* < .05 for all analyses. No formal a priori sample size calculation was performed; however, a sample of 100 participants (50 per group) provided sufficient power for detecting medium-to-large effect sizes (Cohen *d* ≥ 0.5) with 80% power at a two-sided significance level of 0.05.

## 3. Results

### 3.1. Participant characteristics

**Figure 1.**
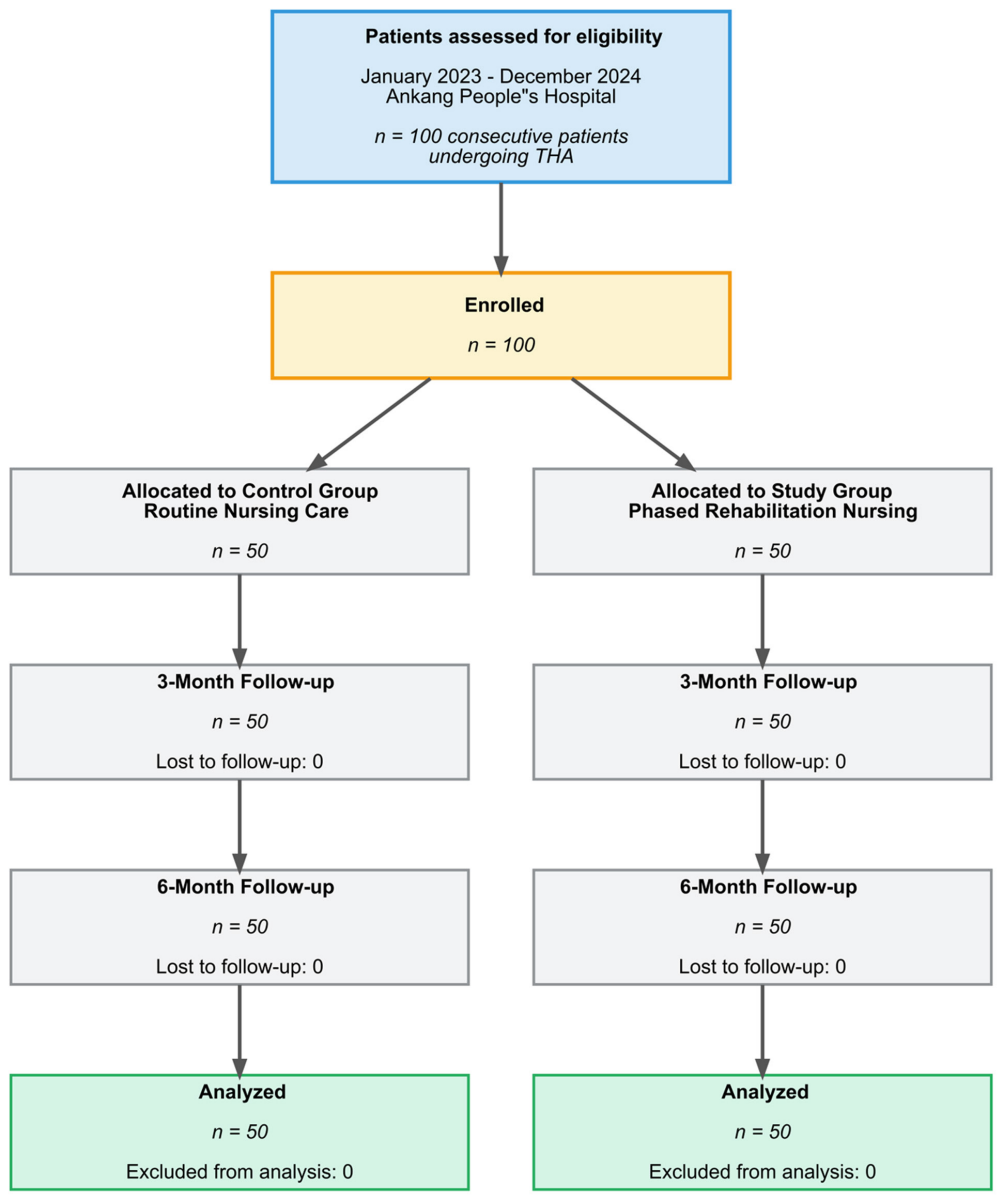
Flow diagram of study participant recruitment and follow-up. THA = total hip arthroplasty.

The study enrolled 100 participants who completed the 6-month follow-up period without loss to follow-up (Fig. 1). Baseline demographic and clinical characteristics demonstrated adequate comparability between groups, with no statistically significant differences between groups (Table 1). The cohort had a mean age approaching 70 years, with a slight male predominance (55%). Primary diagnoses leading to THA were distributed similarly between groups, with femoral neck fractures representing approximately half of all cases (50% in the control group and 52% in the study group), followed by avascular necrosis of the femoral head (30% in the control group and 28% in the study group) and hip osteoarthritis (20% in both groups).

### 3.2. Pain outcomes

**Figure 2.**
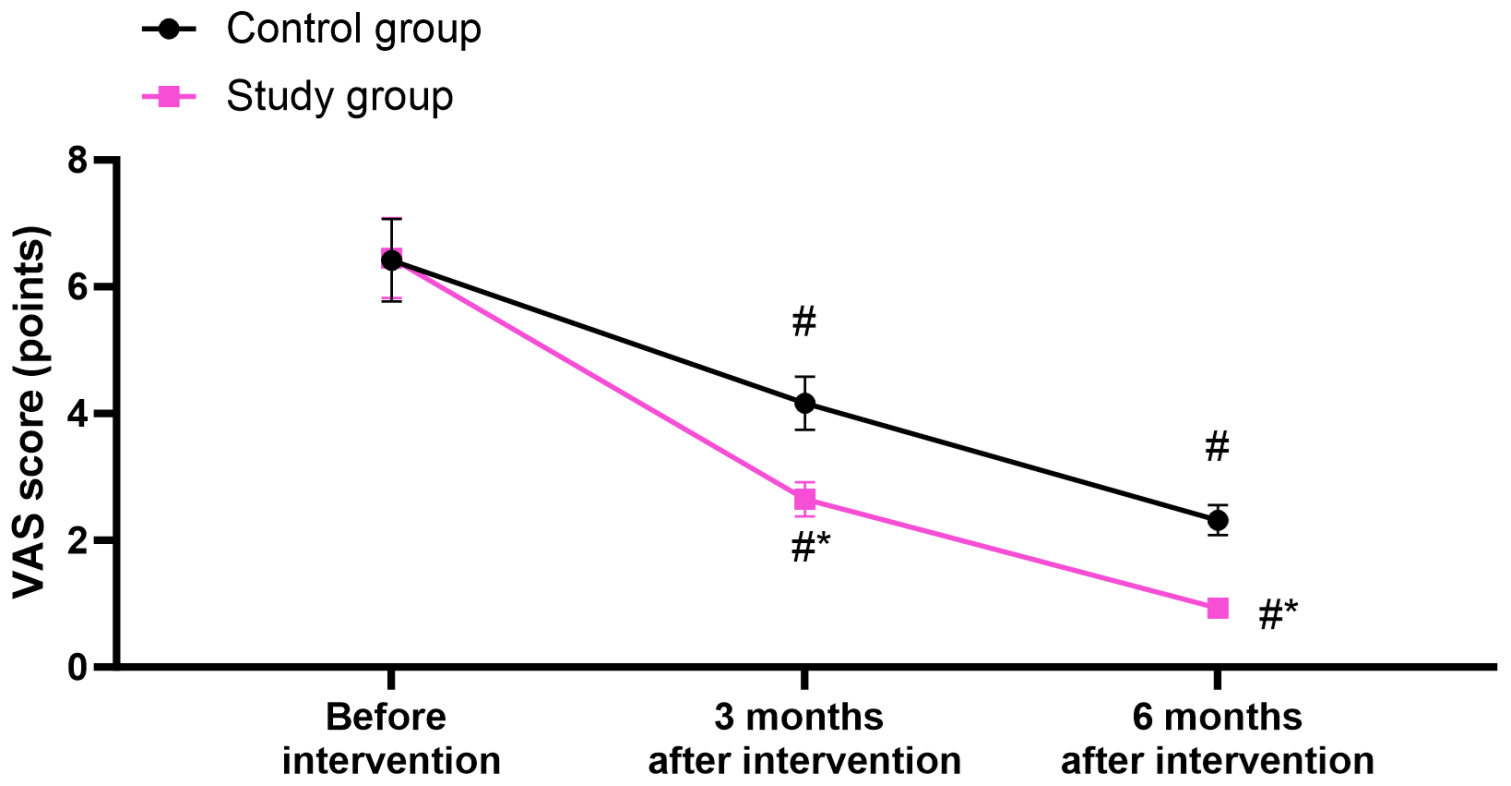
Temporal changes in pain intensity assessed using the Visual Analog Scale (VAS) following total hip arthroplasty. Comparison of pain scores between the control group (routine nursing care, n = 50) and the study group (phased rehabilitation nursing, n = 50) measured at baseline (preoperative), 3 months, and 6 months post-intervention. #*P* < .05 compared with baseline within the same group (paired *t* test); *P < .05 compared with the control group at the same time point (independent samples *t* test). RM-ANOVA: time-by-group interaction, *P* < .001. RM-ANOVA = repeated-measures analysis of variance, VAS = Visual Analog Scale.

Analysis of pain intensity revealed significant improvements in both groups over the 6-month observation period, with the PR group demonstrating superior pain reduction (Fig. 2). At baseline, the VAS scores were comparable between the groups, indicating a similar preoperative pain burden. Three months post-intervention, both groups showed marked pain reduction from baseline (*P* < .05); however, the study group achieved significantly lower pain scores than the control group (*P* < .05). This differential effect persisted and amplified at the 6-month assessment, where the PR group maintained substantially lower VAS scores than the control group (*P* < .05). RM-ANOVA confirmed a significant time-by-group interaction effect (*F* = 15.42, *P* < .001), indicating that the trajectory of pain reduction differed significantly between groups, with the PR group exhibiting a steeper and more sustained decline in pain intensity.

### 3.3. Hip function recovery

**Figure 3.**
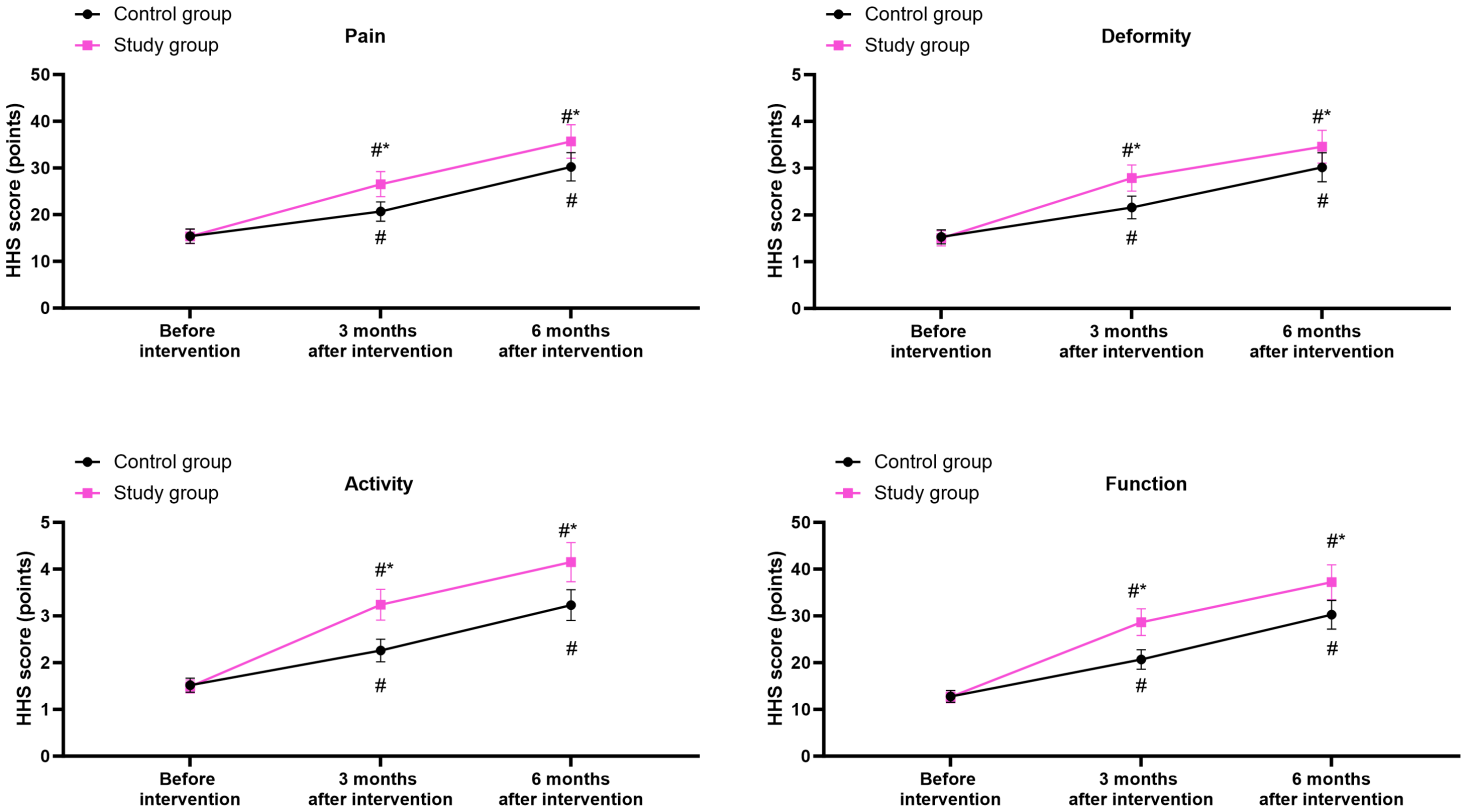
Recovery of hip function as assessed by the Harris Hip Score (HHS) over 6 months after total hip arthroplasty. Longitudinal assessment of hip function in the control group (routine nursing care, n = 50) versus the study group (phased rehabilitation nursing, n = 50) at baseline (preoperative), 3 months, and 6 months post-intervention. #*P* < .05 compared with baseline within the same group (paired *t* test); *P < .05 compared with the control group at the same time point. RM-ANOVA: time-by-group interaction, *P* < .001. HHS = Harris Hip Score, RM-ANOVA = repeated-measures analysis of variance.

Functional recovery patterns, as measured by HHS, demonstrated progressive improvement in both cohorts, with notable advantages for the PR protocol (Fig. 3). Baseline HHS scores indicated comparable functional impairment between groups prior to intervention. At 3 months, while both groups showed significant functional gains from baseline (*P* < .05), the study group achieved superior HHS scores compared with the controls (*P* < .05). This functional advantage persisted and expanded at 6 months, with the PR group maintaining significantly higher functional scores (*P* < .05). RM-ANOVA revealed a significant time-by-group interaction effect (*F* = 12.87, *P* < .001), substantiating the differential recovery trajectories between groups.

### 3.4. PO complications

Surveillance for PO complications revealed a notable reduction in adverse events among patients who received PR nursing (Table 2). The overall complication rate was significantly lower in the study group (2.00%) than in the control group (16.00%; *P* = .01). Specifically, the control group experienced 3 cases of joint stiffness (6.00%), 3 cases of deep venous thrombosis (6.00%), and 2 pressure injuries (4.00%). In contrast, the PR group reported only a single case of joint stiffness (2.00%), with no thrombotic events or pressure injuries. However, individual complication types did not reach statistical significance when analyzed separately (all *P* > .05, Fisher exact test), and the small absolute number of events (9 total across both groups) necessitates cautious interpretation of these findings.

**Table 2:** Incidence of postoperative complications during the 6-month follow-up period.

Complications	Control group (n = 50)	Study group (n = 50)	*P*-value
Individual complications, n (%)			
Joint stiffness	3 (6.0)	1 (2.0)	.31*
Deep venous thrombosis	3 (6.0)	0 (0.0)	.08*
Pressure injury	2 (4.0)	0 (0.0)	.15*
Total patients with complications, n (%)	8 (16.0)	1 (2.0)	.01†

Joint stiffness was defined as range of motion insufficient for activities of daily living; deep venous thrombosis confirmed by Doppler ultrasound; pressure injuries graded according to National Pressure Injury Advisory Panel guidelines.

* Fisher’s exact test was used for individual complications due to small cell counts.

† Chi-square test was used for overall complication rate.

### 3.5. Quality of life improvements

**Figure 4.**
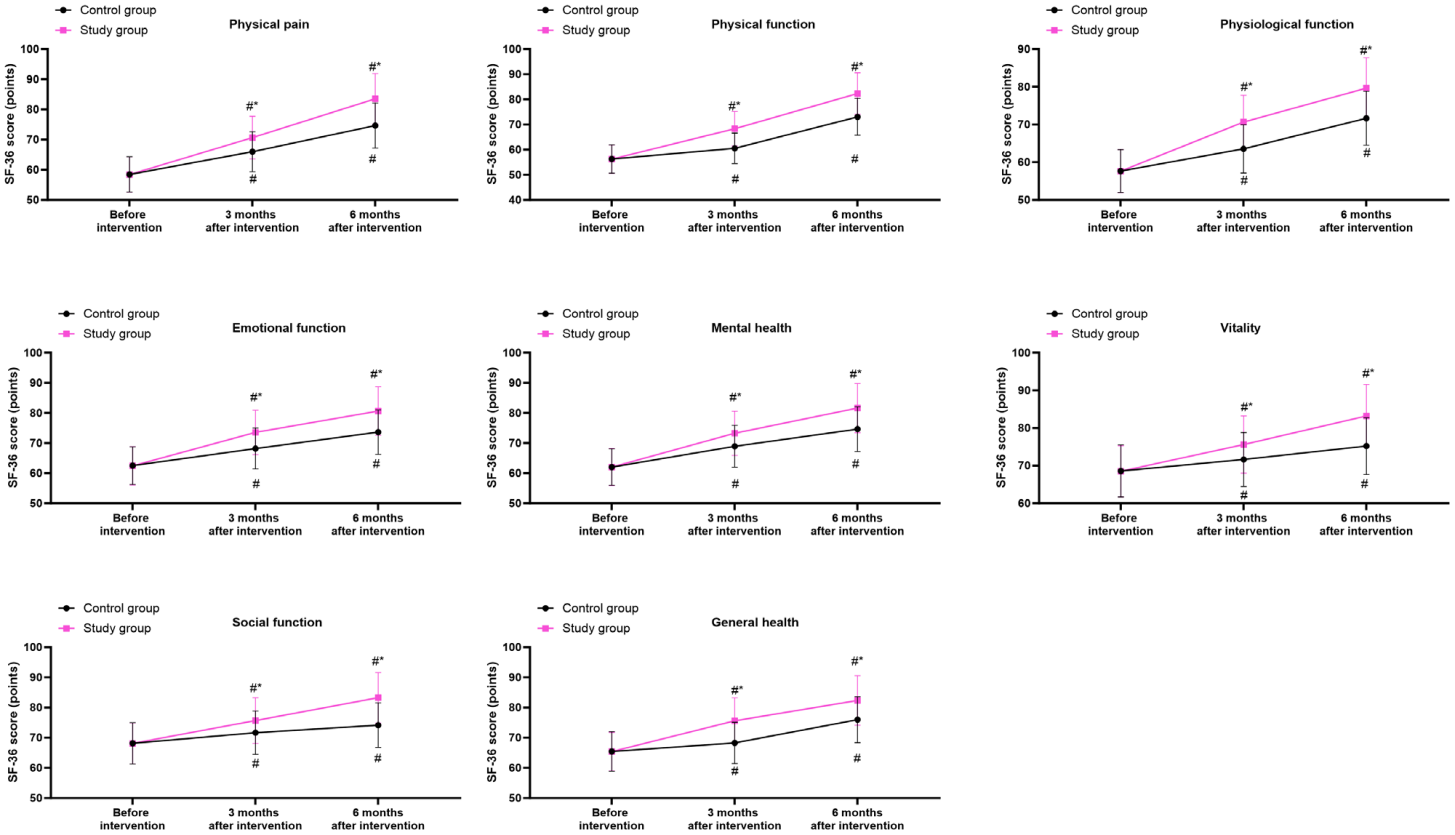
Quality of life outcomes measured using the Short-Form 36 (SF-36) domains after total hip arthroplasty. Comprehensive assessment of 8 health-related quality of life domains comparing the control group (routine nursing care, n = 50) and the study group (phased rehabilitation nursing, n = 50) at baseline, 3 months, and 6 months post-intervention. Scores range from 0 to 100, with higher scores indicating better quality of life. #*P* < .05 compared with baseline within the same group (paired *t* test); *P < .05 compared with the control group at the same time point. RM-ANOVA: significant time-by-group interactions across all domains (all *P* < .05). RM-ANOVA = repeated-measures analysis of variance, SF-36 = Short-Form 36.

A comprehensive quality-of-life assessment using the SF-36 revealed substantial improvements across all 8 domains for both groups, with consistently superior outcomes in the PR cohort (Fig. 4). Three months post-intervention, patients who received PR demonstrated significantly higher scores across physical functioning, bodily pain, role limitations, emotional well-being, social functioning, energy levels, mental health, and general health perceptions than the controls (all *P* < .05). These quality-of-life advantages were maintained and enhanced in several domains at the 6-month assessment. RM-ANOVA demonstrated significant time-by-group interaction effects across all SF-36 domains (all *P* < .05), confirming that the PR group experienced more pronounced improvement trajectories.

### 3.6. Functional independence in daily activities

**Figure 5.**
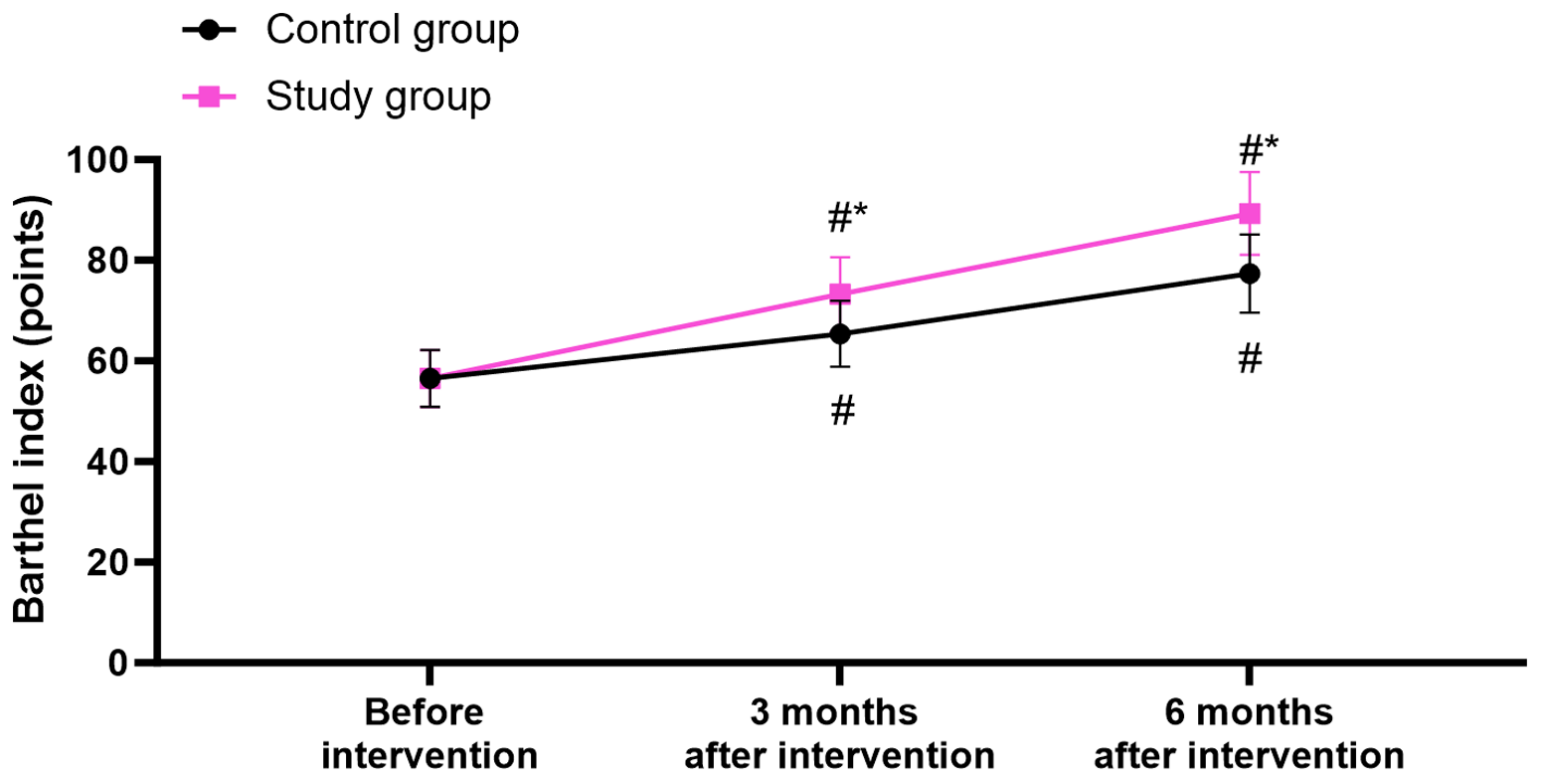
Functional independence in activities of daily living measured using the Barthel Index following total hip arthroplasty. Temporal changes in functional independence comparing the control group (routine nursing care, n = 50) and the study group (phased rehabilitation nursing, n = 50) were assessed at baseline (preoperative), 3 months, and 6 months post-intervention. #*P* < .05 compared with baseline within the same group (paired *t* test); *P < .05 compared with the control group at the same time point. RM-ANOVA: time-by-group interaction, *P* < .001. RM-ANOVA = repeated-measures analysis of variance.

Assessment of activities of daily living through the Barthel Index showed progressive improvement in functional independence for both groups, with accelerated recovery in the PR cohort (Fig. 5). Baseline Barthel scores indicated comparable functional dependency between groups. Following the intervention, both groups demonstrated significant improvements at 3 and 6 months compared with baseline (*P* < .05). However, the PR group achieved significantly higher Barthel Index scores at both follow-up time points compared with the control group (*P* < .05). The time-by-group interaction effect was significant on RM-ANOVA (*F* = 10.35, *P* < .001).

### 3.7. Self-care capacity development

**Figure 6.**
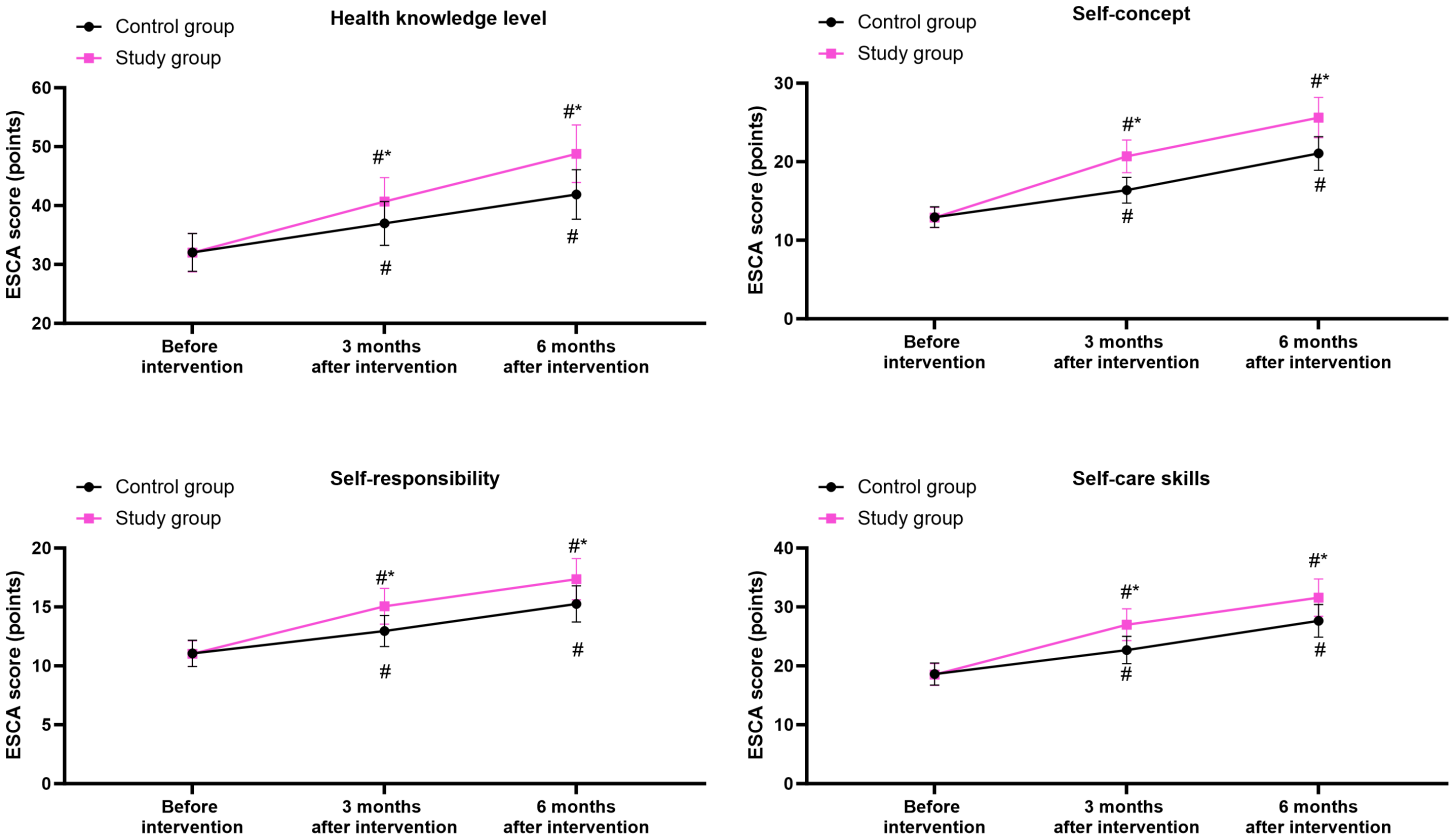
Self-care capacity development was assessed using the Exercise of Self-care Agency Scale (ESCA) following total hip arthroplasty. Longitudinal evaluation of self-care abilities across 4 domains comparing the control group (routine nursing care, n = 50) and the study group (phased rehabilitation nursing, n = 50) at baseline, 3 months, and 6 months post-intervention. Total scores range from 0 to 100, with higher scores indicating greater self-care capacity. #*P* < .05 compared with baseline within the same group (paired *t* test); *P < .05 compared with the control group at the same time point. RM-ANOVA: significant time-by-group interactions for all domains (all *P* < .05). ESCA = Exercise of Self-care Agency Scale, RM-ANOVA = repeated-measures analysis of variance.

Evaluation of self-care abilities using the ESCA revealed substantial improvements across all 4 domains (health knowledge level, self-concept, self-responsibility, and self-care skills) in both groups, with superior outcomes in the PR cohort (Fig. 6). At 3 months, the study group demonstrated significantly higher scores across all ESCA dimensions compared with the controls (*P* < .05), reflecting enhanced patient empowerment and self-management capabilities. These advantages persisted through the 6-month assessment, suggesting that the structured educational and skill-building components of PR nursing fostered sustained improvements in patients’ ability to manage their recovery independently. RM-ANOVA confirmed significant time-by-group interactions for all 4 ESCA domains (all *P* < .05).

### 3.8. Treatment adherence and exercise compliance

Analysis of exercise compliance revealed substantially higher adherence rates in the PR group (96.00%) than in the routine care (RC) group (80.00%; *P* = .01), as shown in Table 3. Complete compliance with the prescribed exercise protocols was achieved by 50% of study group participants versus 40% of controls, whereas partial compliance was observed in 46% and 40% of the respective groups. Notably, noncompliance was markedly reduced in the PR group (4.00%) compared with that in the RC group (20.00%), suggesting that the structured, progressive nature of the intervention enhanced patient engagement and adherence to rehabilitation protocols.

**Table 3:** Exercise compliance assessment at 6-month follow-up.

Compliance category	Control group (n = 50)	Study group (n = 50)	Definition
Complete compliance, n (%)	20 (40.0)	25 (50.0)	Strict adherence to prescribed exercise protocol without deviation
Partial compliance, n (%)	20 (40.0)	23 (46.0)	Occasional deviations from prescribed protocol (<2 missed sessions/wk)
Noncompliance, n (%)	10 (20.0)	2 (4.0)	Frequent protocol omissions (≥2 missed sessions/wk)
Overall compliance rate, n (%)	40 (80.0)	48 (96.0)	Complete + partial compliance
		**χ** ^**2**^ ** = 6.06, *P* = .01**	

Compliance was assessed through patient self-report diaries and therapist documentation during follow-up visits. The overall compliance rate represents the proportion of patients achieving complete or partial compliance with the rehabilitation protocol.

### 3.9. Patient satisfaction with nursing care

Patient satisfaction assessment demonstrated significantly higher satisfaction rates with PR nursing (94.00%) than with RC (78.00%, *P* = .02), as detailed in Table 4. The proportion of patients reporting being “very satisfied” was higher in the study group (48.00%) than in the controls (40.00%), while dissatisfaction rates were substantially lower (6.00% vs 22.00%). These findings reflect patients’ positive experiences with the comprehensive, individualized approach of PR nursing, encompassing not only physical recovery but also educational support, psychological encouragement, and continuity of care through structured follow-up.

**Table 4:** Patient satisfaction with nursing care at 6-month follow-up.

Satisfaction level	Control group (n = 50)	Study group (n = 50)	Score range
Very satisfied, n (%)	20 (40.0)	24 (48.0)	90–100 points
Satisfied, n (%)	19 (38.0)	23 (46.0)	80–89 points
Dissatisfied, n (%)	11 (22.0)	3 (6.0)	<80 points
Overall satisfaction rate, n (%)	39 (78.0)	47 (94.0)	Very satisfied + satisfied
		**χ** ^**2**^ ** = 5.31, *P* = .02**	

Satisfaction was assessed using a validated 100-point institutional questionnaire covering multiple domains of nursing care, including pain management, education, emotional support, and care coordination. The overall satisfaction rate represents the proportion of patients reporting satisfied or very satisfied ratings.

## 4. Discussion

This prospective quasi-experimental study demonstrates that PR nursing significantly improves multiple outcomes following THA compared with RC. Patients who received the structured intervention experienced reduced pain, enhanced hip function recovery, fewer complications, and improved quality of life over 6 months. These benefits extended to functional independence, self-care capacity, exercise adherence, and patient satisfaction.

Our findings of superior pain control and functional improvement align with established rehabilitation principles for THA recovery.^[19]^ The significant reduction in pain scores and enhanced hip function in the PR group likely resulted from the systematic progression of therapeutic interventions. Although routine nursing provides standard PO care addressing multiple aspects of recovery, it may not adequately accommodate individual differences or address stage-specific rehabilitation needs.^[20–22]^ The structured, progressive nature of PR appears to optimize recovery trajectories through targeted interventions matched to each recovery stage.

The effectiveness of PR nursing can be attributed to several interconnected mechanisms operating across different recovery phases. During the early mobilization phase (days 1–4), the initial focus on muscle stimulation and controlled range-of-motion exercises promotes circulation while protecting the surgical site.^[23]^ Lower extremity muscle compression enhances venous return and reduces edema, whereas ankle exercises prevent stiffness and thrombosis risk.^[24]^ These early interventions establish the foundation for progressive mobility without compromising surgical integrity. As patients transition to the intermediate phase (days 5–7), progressive weight-bearing and position changes enhance proprioception and muscle control. The systematic progression from supported positions to standing prevents deconditioning while building patient confidence. Therapeutic heat application through Teding Diancibo Pu lamp therapy^[25]^ may enhance local circulation and tissue healing, although further research is needed to confirm this mechanism. The advanced mobility phase, beginning in week 2, involves progression from walker to crutch assistance and eventual stair navigation, representing the achievement of functional milestone.^[26]^ This structured advancement ensures that patients develop the necessary strength and coordination before attempting complex movements, potentially explaining the reduced complication rates observed.

In addition to physical interventions, the phased dietary progression from easily digestible foods to calcium-rich nutrition supports wound healing and bone integration. During weeks 1 to 2, an emphasis on anti-inflammatory foods may reduce PO edema, whereas weeks 3 to 4 focus on supporting bone metabolism. This nutritional timing aligns with the physiological healing stages; however, specific dietary effects on THA recovery warrant further investigation.^[27]^ Furthermore, comprehensive discharge education and structured follow-up likely contributed to the high exercise compliance rates (96% vs 80%). By providing clear activity guidelines and maintaining regular contact through clinic visits and remote monitoring, the intervention sustained patient engagement beyond hospitalization. This continuity of care model addresses a critical gap in THA rehabilitation, in which many patients struggle with adherence after discharge.

However, several alternative explanations for the observed findings merit consideration. The superior outcomes in the study group may be partially attributable to the Hawthorne effect, whereby patients receiving a novel, more intensive intervention may report more favorable outcomes due to heightened attention and expectation rather than the specific therapeutic content of the protocol alone. Additionally, the increased frequency of contact and the personalized nature of the PR protocol inherently provide more nursing attention and interaction time compared with RC. This differential attention, independent of the specific rehabilitation components, could have contributed to improved patient satisfaction and self-reported outcomes, such as pain and quality of life. The absence of blinding in this study means that both patients and nursing staff were aware of group assignment, introducing the possibility of performance and detection bias, particularly for subjective outcome measures.

Regarding the complication findings, although the overall complication rate was significantly lower in the study group (2% vs 16%, *P* = .01), the absolute number of complication events was small (9 total events across both groups). Therefore, the apparently substantial relative reduction should be interpreted with caution, as such effect magnitudes are inherently unstable when derived from small event counts. Individual complication types did not achieve statistical significance when analyzed separately, and the study was not specifically powered to detect differences in individual complication rates. Adequately powered multicenter studies with complication rates as a primary endpoint are necessary to confirm any protective effect of the PR protocol against specific PO complications.

Our findings align with and extend previous research on rehabilitation after THA. Che et al demonstrated that exercise prescription-based rehabilitation improved hip function and reduced complications in elderly THA patients.^[28]^ Similarly, Gilbey et al showed that structured exercise enhanced early functional recovery.^[29]^ Our study adds to this evidence by demonstrating that a nursing-led phased approach can achieve promising outcomes through systematic progression of interventions, although the nonrandomized design precludes definitive causal conclusions.

This study has several important limitations that should be acknowledged. First, the quasi-experimental design with ward-based allocation, while pragmatic, introduces the possibility of selection bias. Although baseline characteristics were comparable between the groups, unmeasured confounders related to ward environment, nursing team characteristics, or patient preferences cannot be excluded. Second, the single-center design conducted at a single hospital in Shaanxi Province limits the generalizability of our findings to other healthcare settings with potentially different resources, patient populations, and nursing practices. Third, the sample size of 100 participants, while providing adequate power for detecting medium-to-large effects on primary outcomes, limits the precision of estimates for secondary outcomes and precludes meaningful subgroup analyses. Fourth, several outcome measures, including exercise compliance and patient satisfaction, relied on self-reporting, which is susceptible to social desirability bias and recall inaccuracy. Fifth, the absence of blinding, which is inherently difficult in nursing intervention studies, may have influenced both patient-reported outcomes and nursing behavior. Sixth, the 6-month follow-up period, while capturing the critical early recovery trajectory, does not establish whether benefits persist over the longer term. Finally, no formal a priori sample size calculation was conducted, representing a methodological limitation that future studies should address.

Future research should include multicenter randomized controlled trials to confirm these findings in diverse settings. Investigating optimal phase durations and transition criteria could refine the protocol. Economic analysis would clarify cost-effectiveness, whereas qualitative studies could explore patient experiences and preferences. Component analysis studies that isolate the effects of individual protocol elements (e.g., structured mobilization vs nutritional guidance vs follow-up intensity) would help identify the active ingredients of the intervention and facilitate more targeted implementation.

## 5. Conclusion

PR nursing significantly improves recovery after THA through systematic and progressive interventions tailored to recovery stages. This approach reduces pain and complications while enhancing hip function, quality of life, functional independence, and self-care capacity. The structured protocol also improves exercise adherence and patient satisfaction. However, as these findings were derived from a single-center quasi-experimental design with a relatively modest sample size, they should be interpreted as preliminary evidence that warrants confirmation through rigorously designed multicenter randomized controlled trials before broad clinical implementation can be recommended.

## Author contributions

**Conceptualization:** Xin Wang, Dan Wang.

**Data curation:** Xin Wang, Dan Wang.

**Formal analysis:** Xin Wang, Dan Wang.

**Funding acquisition:** Dan Wang.

**Project administration:** Dan Wang.

**Supervision:** Dan Wang.

**Validation:** Dan Wang.

**Investigation:** Xin Wang, Dan Wang.

**Methodology:** Xin Wang, Dan Wang.

**Resources:** Xin Wang, Dan Wang.

**Software:** Xin Wang, Dan Wang.

**Visualization:** Xin Wang, Dan Wang.

**Writing – original draft:** Xin Wang, Dan Wang.

**Writing – review & editing:** Xin Wang, Dan Wang.
